# Playing Jekyll and Hyde—The Dual Role of Lipids in Fatty Liver Disease

**DOI:** 10.3390/cells9102244

**Published:** 2020-10-06

**Authors:** Martijn R. Molenaar, Louis C. Penning, J. Bernd Helms

**Affiliations:** 1 Department of Biomolecular Health Sciences, Faculty of Veterinary Medicine, Utrecht University, 3584 CM Utrecht, The Netherlands; m.r.molenaar@uu.nl (M.R.M.); j.b.helms@uu.nl (J.B.H.); 2 Department of Clinical Sciences of Companion Animals, Faculty of Veterinary Medicine, Utrecht University, 3584 CM Utrecht, The Netherlands

**Keywords:** lipid droplets, lipid metabolism, hepatocytes, hepatic stellate cells, non-alcoholic fatty liver disease, DGAT2

## Abstract

Lipids play Jekyll and Hyde in the liver. On the one hand, the lipid-laden status of hepatic stellate cells is a hallmark of healthy liver. On the other hand, the opposite is true for lipid-laden hepatocytes—they obstruct liver function. Neglected lipid accumulation in hepatocytes can progress into hepatic fibrosis, a condition induced by the activation of stellate cells. In their resting state, these cells store substantial quantities of fat-soluble vitamin A (retinyl esters) in large lipid droplets. During activation, these lipid organelles are gradually degraded. Hence, treatment of fatty liver disease is treading a tightrope—unsophisticated targeting of hepatic lipid accumulation might trigger problematic side effects on stellate cells. Therefore, it is of great importance to gain more insight into the highly dynamic lipid metabolism of hepatocytes and stellate cells in both quiescent and activated states. In this review, part of the special issue entitled “Cellular and Molecular Mechanisms underlying the Pathogenesis of Hepatic Fibrosis 2020”, we discuss current and highly versatile aspects of neutral lipid metabolism in the pathogenesis of non-alcoholic fatty liver disease (NAFLD).

## 1. Introduction

Chronic liver diseases affect hundreds of millions of people worldwide. Non-alcoholic fatty liver disease (NAFLD) represents a wide range of liver diseases that are all caused by stimuli other than excessive alcohol consumption, covering simple steatosis (the accumulation of fat in hepatocytes), non-alcoholic steatohepatitis (NASH), fibrosis and its more severe form cirrhosis. Moreover, NAFLD is a risk factor for the development of hepatocellular carcinoma, cardiovascular diseases and type 2 diabetes (reduced insulin sensitivity). Currently, NAFLD is viewed as the most frequent cause of chronic liver failure, with a prevalence of approximately 20–30% in the Western world, and is expected to rise significantly in view of the increased number of obese people worldwide [1]. NAFLD affects the quality of life of almost 2 billion people worldwide [2]. In this review, we focus on the complex lipid dynamics in NAFLD. Signaling pathways that mediate the livers’ inflammatory status caused by lipid-laden hepatocytes in steatohepatitis are reviewed elsewhere [3].

The estimated number of lipid compounds (ranging between 30,000 and 180,000) exceeds by far—at least theoretically—the number of genes reported in mammals [4,5]. This high number hints to the existence of a highly regulated and complex system to fulfill the myriad of functions of lipids. Furthermore, lipids are—by definition—poorly soluble in water, posing some physical-chemical constraints since biological systems are dominated by aqueous environments.

Virtually all eukaryotic cells have the capacity to store lipids in specific organelles, the so-called lipid droplets (LDs). LDs are organelles specialized in the storage of hydrophobic components [6,7,8]. The organelle consists of a hydrophobic core containing neutral lipids—triacylglycerols (TAGs), cholesteryl esters (CEs), acylceramides and/or retinyl esters (REs)—surrounded by a phospholipid monolayer and proteins that facilitates structure, integrity and several enzymatic activities responsible for synthesis or degradation of the LD content [6,7,8]. The most abundant LD proteins are perilipins, of which five family members are described, encoded by the genes *Plin 1–5* [9,10,11,12]. The formation of LDs occurs in the ER, driven by a biophysical process in which neutral lipids segregate from phospholipid bilayers and emerge from the cytosolic leaflet of the ER-membrane [7]. How this process is regulated is still poorly understood. Several proteins (families) have been implicated to be players in this process—FIT proteins (fat storage-inducing transmembrane protein 1 and 2), seipin, and, more recently, LDAF (lipid droplet assembly factor 1, previously known as TMEM159) [13,14,15,16].

Some cell types are specialized and optimally suited for the storage of neutral lipids. Examples include adipocytes, Leydig cells, and hepatic stellate cells (HSCs). In adipocytes, the main cells in adipose tissue, TAGs are stored to secure long-term supply of fatty acids for energy production. In Leydig cells, lipid droplets provide sufficient amounts of cholesterol for the synthesis of steroid hormones. Liver-resident HSCs are specialized in the storage of vitamin A as REs, a fat-soluble vitamin required for, e.g., vision, development, and reproduction. These cells are important players in NAFLD and will be discussed in more detail later in this review.

**Figure 1:**
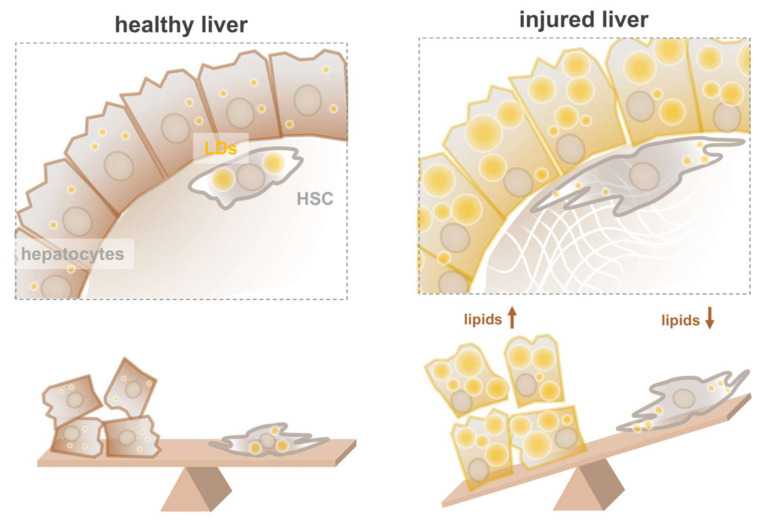
The “fat” paradox in liver. How to specifically target fat accumulation of hepatocytes? Simplified cartoons of (**left**) healthy liver with normal hepatocytes flanked by a quiescent hepatic stellate cell (HSC) with large lipid droplets (LDs), and (**right**) injured liver with lipid-filled hepatocytes flanked by activated HSCs devoid of large LDs.

In order to perform the various functions of a liver, such as biotransformation, coagulation factor production, and lipid homeostasis, the organ harbors various cell types. The most abundant cells in the liver are the hepatocytes, accounting for approximately 60–70% of all liver cells, whereas other cell types are less abundant, e.g., HSCs (vitamin A storage, 5–15%) [17], Kupffer cells (liver macrophages, 10–15%) [18], cholangiocytes (bile duct cells, 3–5%) [19], and liver sinusoidal endothelial cells (blood vessel lining, 15–20%) [20]. In a healthy liver, hepatocytes store limited amounts of neutral lipids in LDs. In contrast, HSCs contain large LDs, responsible for the storage of most of the vitamin A in the body [17,21]. When livers accumulate excessive amounts of lipids within hepatocytes, liver function is hindered. Paradoxically, in response to hepatocyte lipid accumulation, HSCs lose their large vitamin A-laden LDs and “transactivate” into an extracellular matrix (ECM)-producing phenotype, a hallmark of liver fibrosis [17,21]. Therefore, strategies to keep HSCs in an LD-rich state and at the same time lower LDs in hepatocytes are likely to be instrumental in the prevention or reversal of liver fibrosis. This approach might, however, also negatively affect the lipogenic state of HSCs. This “fat” paradox (Figure 1) [22] often complicates the effective treatment of liver disease. To provide some leads, we will present an overview of lipid metabolism and LD dynamics in the liver, followed by a description of the key players in lipid metabolism in hepatocytes and HSCs. We end with discussing recent promising models to better study the mechanisms of liver disease and screen for potential therapeutic drugs in vitro.

## 2. Lipids and Liver Diseases

Lipids are essential structural components of cellular membranes, energy-storage molecules, signaling mediators in and between cells, and regulators in inflammation. The liver is the central organ in the regulation of lipid homeostasis in vertebrates, including production of lipids (de novo lipogenesis, DNL), storage of lipids as TAGs in LDs, and secretion of lipids by albumin, retinol-binding proteins, and lipoproteins. This last class is subdivided into at least three major categories: very-low-density lipoproteins (VLDL), low-density lipoproteins (LDLs), and high-density lipoproteins (HDLs). As a result, it is not a surprise that malfunction of one of these lipid-related processes leads to liver pathology. Liver functions are mainly performed by the parenchymal cells of the liver—the hepatocytes. The most prominent lipid-associated liver disease—with increasing prevalence worldwide—is NAFLD, representing an accumulation of neutral lipids in hepatocytes. Strikingly, 10–30% of the NAFLD patients progress towards NASH, of which a significant number (20–30%) progress into the cirrhotic stage of the disease [23], highlighting the importance of preventing or reversing early hepatic lipid accumulation. Importantly, there is an increasing awareness that NAFLD should not viewed as isolated liver disease. Rather, NAFLD is associated with features of metabolic syndrome, a systemic disease that is represented by a cluster of conditions that occur together, thus increasing the risk of heart disease, stroke, type 2 diabetes and obesity. Hence, it has recently been proposed that the term metabolic-associated fatty liver disease (MAFLD) better reflects current knowledge [24,25].

## 3. Lipid Accumulation in Hepatocytes

Although the liver plays a central role in the lipid homeostasis of the body, healthy hepatocytes store only limited amounts of neutral lipids. As the body’s central lipid-distribution hubs, however, hepatocytes are susceptible to anomalies in any of the systems that are involved in lipid homeostasis. TAGs are used as indicators for the severity of NAFLD, but TAGs are by themselves not hepatotoxic. This in contrast to other lipids that accumulate in fatty liver, such as free fatty acids (FFAs), cholesterol, oxysterols, diacylglycerol, and phospholipids [26]. TAG synthesis might be a strategy to prevent toxic FFA accumulation in hepatocytes [27]. We will discuss this possibility in more detail later in this review.

**Figure 2:**
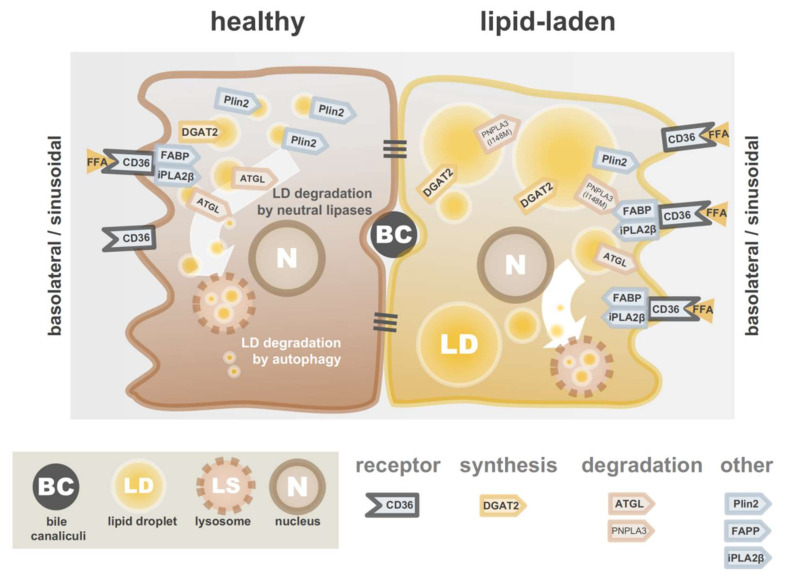
Lipid droplet dynamics in hepatocytes. Cartoon of healthy (**left**) and lipid-laden (**right**) hepatocytes showing key players in LD synthesis, stabilization and degradation. ATGL, adipose triglyceride lipase; BC, bile canaliculi; DGAT2, diacylglycerol acyltransferase 2; FABP, FA-binding protein; iPLA2β, calcium-independent membrane phospholipase A2; LDs, lipid droplets; LS, lysosome; N, nucleus; PLIN2, perilipin 2; PNLPLA3, Patatin-like phospholipase domain-containing protein 3.

Excessive TAG accumulation in hepatocytes can be either the result of (i) overload from exogenous sources, (ii) endogenous overproduction, (iii) insufficient lipid catabolism, (iv) impaired secretion, or a combination of these factors. Major external sources of lipids are free fatty acids from adipocytes (responsible for approximately 60% of NAFLD patient livers) and dietary intake (ca. 14%). In addition, excessive *de novo* lipogenesis accounts for the remaining 26% in NAFLD patient livers [28]. During an imbalance in the lipid homeostasis caused by either one of the mechanisms mentioned above, hepatocytes gradually accumulate neutral lipids. At some point, the LDs storing these lipids coalesce and can eventually become so large that they fill most of the hepatocyte’s cytoplasm. This phenomenon often coincides with a characteristic feature of NAFLD: hepatocytes with increased diameters (1.5 to 2-fold), rarefied cytoplasms and cytoskeletal rearrangements, a feature referred to as “ballooning” [29,30]. In addition, the composition of the various lipids within and surrounding the LDs changes upon high-fat diet [31]. For instance, the increase in TAGs is mainly driven by the incorporation of saturated or monounsaturated fatty acids. Moreover, total amounts of diacylglycerols, lysolipids and sphingolipids increase [31]. The variety in drivers of lipid accumulation are summarized in Figure 2 and offers several potential strategies to limit the overload in hepatocytes, which will be covered below.

### 3.1. Limiting Overload of Lipids from Exogenous Sources

Non-esterified fatty acids (NEFAs) from adipocytes are taken up by hepatocytes via the surface protein CD36 [32], in concert with caveolin-1 and plasma membrane (PM) FA-binding protein (FABP_PM_) [33]. Fatty acid uptake by rat hepatocytes was found to be reduced by FABP_PM_-neutralizing antibodies [34,35]. Furthermore, expression of CD36 was shown to be higher in obese patients with NAFLD [36], and reduced lipid accumulation after interfering with CD36 has been reported previously [37,38]. Mice on a high-fat diet and lacking hepatocyte-specific CD36 presented decreased liver lipid accumulation and improved insulin sensitivity compared to mice with normal CD36 expression [37]. In miR-29aTg mice (expressing transgenic miRNA-29a), both expression of CD36 and fat diet-induced hepatic lipid accumulation was reduced. Moreover, other factors indicative for steatohepatitis were reduced: pro-inflammatory cytokines such as IL-6 and MCP1, and markers for the epithelial mesenchymal transition such as Snail and Vimentin [38]. This observation is in line with other reports describing positive effects of miRNA-29a on liver fibrosis [39,40]. Interestingly, CD36 was also implicated as a negative regulator of lipophagy in hepatocytes (lipophagy is discussed later) [41]. Together, reduced CD36-mediated lipid uptake reduces lipid accumulation in hepatocytes.

Fatty acid influx rates are also affected by calcium-independent membrane phospholipase A2 (iPLA2β). Hepatocytes isolated from iPLA2β-deficient mice showed a 56% decrease in fatty acid influx compared to wild-type mice [42]. The protein itself does not display fatty acid binding affinity but might interact with the fatty acid binding complex (with CD36, caveolin-1 and FABP).

### 3.2. Limiting Endogenous Overproduction of Lipids

De novo synthesis of lipids (DNL) is the second most important source of intrahepatic lipid accumulation. In this process, the liver converts excess carbohydrates into lipids. Hence, an effective way to minimize DNL is to limit sugar intake. Although glucose is the primary substrate for DNL, fructose was shown to be highly lipogenic, possibly by bypassing a critical regulatory step—phosphofructokinase-1 (PFK-1)—in the glycolysis [43,44]. Several transcription factors regulate DNL, including as sterol regulatory element binding protein 1 (SREBP1), carbohydrate response element binding protein (ChREBP) and liver X receptors [45,46,47].

Interestingly, DNL is upregulated in NAFLD patients. In one study, DNL was approximately doubled in patients suffering from NAFLD in comparison with healthy individuals [48]. In another study, DNL contributed to 11, 20 or 40% of the total hepatic lipid accumulation in cohorts of healthy, obese or obese-NAFL individuals, respectively [49]. Data from the same study also suggest a link between insulin resistance and rate of DNL in NAFLD [49] as insulin insensitivity promoted DNL. Moreover, weight loss of patients resulted in decreased DNL and hepatic lipid accumulation.

The synthesis of TAGs, the main neutral lipids in LDs, is catalyzed by diacylglycerol acyltransferases (DGATs). In mammals, two enzymes without shared homology, DGAT1 and DGAT2, are described [50]. Although both enzymes utilize the same substrates, diacylglycerol and fatty acid-coA, their functions are thought to be distinct. DGAT1, abundantly expressed in the intestine [51], is suggested to be responsible for the (re)esterfication of exogeneous fatty acids to prevent ER-stress [52]. By contrast, DGAT2 is believed to be the main player in the esterification of de novo synthesized fatty acids [53,54]. In liver, DGAT2 is highly expressed [51]. As discussed earlier, TAG synthesis might prevent the toxic effects of FFA accumulation in hepatocytes [27], and DGAT2 inhibition to treat NAFLD and/or NASH was met with skepticism. Indeed, a study in which DGAT2 of mice on a methionine and choline deficient diet was targeted using antisense oligonucleotides did show decreased TAG accumulation but did not reduce liver inflammation and fibrosis (in fact, liver inflammation increased) [55]. In another study, the decreased levels of plasma TAGs and VLDL apolipoprotein B observed in obese mice treated with DGAT2 inhibitors could not be reproduced in rhesus primates, questioning the potential of DGAT2 inhibition in humans [56]. Nevertheless, several recent studies report more encouraging results [57,58,59]. A recent multicenter, double-blind, randomized, placebo-controlled study with 44 overweight patients revealed a safe and effective inhibition of DGAT2 by antisense oligonucleotides. In this investigation, a significant reduction in liver fat was observed in the treated group. Other lipid parameters including total cholesterol, TAGs and lipoprotein level remained unaffected throughout the treatment period and a follow-up period of almost three months. Most promising was the observation that hepatic inflammation and fibrosis were reduced in patients that responded well to the treatment and showed reduced hepatic triglyceride levels [58]. Another study with healthy human participants treated with orally administered DGAT2 inhibitors showed that this treatment was successful in reducing hepatic lipid levels. Importantly, also this reduction was accompanied by increased markers associated with liver function, as well as a reduction in liver fibrosis [59]. Combined, these studies highlight the potential of DGAT2 as a druggable target for the treatment of NASH.

### 3.3. Promoting Lipid Catabolism and Lipid Secretion

Enhancing the utilization of lipids might be another strategy to reduce hepatic lipid accumulation. For example, beta-oxidation can be upregulated by the activation of carnitine palmitoyl transferase-1 (CPT-1), a mitochondrial enzyme responsible for the formation of acyl carnitines. Indeed, stimulated expression of CPT-1 by pharmaceuticals reduced hepatic lipid levels [60,61,62].

A reduction in hepatic lipid levels by the induction of LD degradation is also possible. LDs in hepatocytes are degraded via two distinct pathways [63]: classical lipolysis or selective autophagy of LDs, also referred to as lipophagy [64]. Recently, it has been shown that interference in either one of the pathway results in distinct LD phenotypes [63]. The inhibition of adipose triglyceride lipase (ATGL), which blocks classical lipolysis, resulted in large LDs, whereas lysosomal inhibition gave rise to smaller LDs. Interestingly, combined inhibition of both pathways also caused larger LDs, suggesting that autophagy plays a role downstream of ATGL lipolysis. Several studies reported dysfunctional autophagy in livers from NAFLD patients [65,66]. It was shown that blocking autophagy results in increased lipid accumulation in liver [67], while activation of the pathway alleviated NAFLD [68,69]. Nevertheless, specific targeting lipophagy in hepatocytes remains challenging, and non-specific activation of autophagy will go hand in hand with undesired side-effects.

Perilipins are thought to protect LDs from degradation [70]. Deletion of Plin2, the most abundant perilipin in hepatocytes, was therefore investigated for its role in hepatocyte lipolysis [71,72]. Plin2-null mice were protected from obesity and showed enhanced lipid hepatic lipid levels as well as improved insulin sensitivity [72]. Liver-specific Plin2 knockout showed similar effects [71]. Interestingly, lipid analysis in these mice revealed that phosphatidylethanolamine (PE) to phosphatidylcholine (PC) conversion mediated by the enzyme phosphatidylethanolamine N-methyltransferase (PEMT) was affected [71]. Higher PE/PC ratios on LDs are associated with liver injury in rats [73]. More recently, the specific role of Plin2 in hepatocytes was studied in more detail by comparing full-body and liver-specific Plin2 deletion. This investigation showed that although both hepatic and extra-hepatic Plin2 are involved in liver steatosis, Plin2 expressed in hepatocytes plays a specific role in immune cell recruitment and fibrogenesis [74].

Recently, a study showed that the breakdown of hepatic LDs and TAG secretion is promoted by insulin [75]. These findings are remarkable, as insulin promotes TAG synthesis and LD formation in, e.g., adipocytes [76]. It was suggested that this mechanism facilitates the controlled release of TAG-filled lipoproteins, which are known to be virtually unchanged during feeding–fasting cyles [77]. When insulin levels raise, phosphatidic acid on hepatic LDs elevates, which in turn triggers kinesin-1 motors to transport LDs to the smooth ER of the hepatocyte’s periphery. Here, LDs are broken down, packaged into lipoproteins and secreted [75].

The lipid secretion via lipoproteins can be regulated to some extent. Although the number of VLDL particles are similar in NAFLD patients and non-patients [78], total TAG-secretion was found to be higher in NAFLD patients due to higher amounts of TAG per particle. Nevertheless, it has been proposed that there is an upper limit in VLDL sizes that can pass the sinusoidal endothelial pores [79], and thus, that there is a maximal capacity of neutral lipid secretion via lipoproteins.

### 3.4. A Reduction in Lipid Accumulation by Other Mechanisms

A common variant (Ile148Met) of Patatin-like phospholipase domain-containing protein 3 (PNPLA3), an ATGL homologue, has been reported to be an important risk factor for hepatic steatosis [80,81,82,83]. The mutant protein was shown to accumulate on the surface of LDs [84] as a result of disrupted ubiquitylation and proteasomal degradation [85,86]. However, how this mutation results in lipid accumulation is not fully understood. Although PNPLA3-Ile148Met has a lower lipase activity than the wild-type protein—which might explain the lipid accumulation—PNPLA3-null mice do not accumulate lipids [87,88]. It has been proposed that the LD-coated PNPLA3 Ile148Met-variant may recruit CGI-58, a factor responsible for decreases lipolysis [84]. Another hypothesis is that the accumulation of the mutant protein on LDs might affect the presence or concentration of other proteins by a mechanism referred to as molecular crowding [89,90]. Combined, enhancing ubiquitylation of PNPLA3-Ile148Met might be a target to limit hepatic lipid accumulation of patients with this mutation, but effective targeting of this variant for the use as clinical therapeutics will be challenging [91].

It should be noted that lipid accumulation in hepatocytes is largely heterogeneous and often dependent on zonation—the distribution of differentially expressed hepatocytes along the lobule axis. For instance, pericentral hepatocytes, cells that are relatively sparse in nutrients and oxygen, are more likely to accumulate lipids [92,93]. Zonation might have implications for targeting strategies. Driven by the advances in RNAseq technology in recent years, single-cell transcriptomics have revealed the zonal gene expression of healthy hepatocytes in great detail [94]. Intriguingly, these zonal gene expression signatures are highly affected in response to high-fat diets [95]: both periportal and pericentral hepatocytes downregulate many of their typical zonal markers. Intriguingly, high-fat diets further upregulate the expression of a number of genes linked to lipid droplet formation in pericentral hepatocytes, providing an explanation for the extreme zonal distribution in lipid accumulation of steatosed livers [95].

Taken together, lipid homeostasis in hepatocytes is the result of a complex interplay between many players, and perturbations in any of those systems give rise to lipid accumulation. Extreme lipid-overload conditions compromise hepatocyte function and trigger a cascade of events, e.g., secretion of stress factors, inflammation of the liver, activation of Kupffer cells, and—covered in the next sections and one that drives fibrosis—activation of HSCs.

## 4. Hepatic Stellate Cells in Healthy and Diseased Liver

HSCs, also referred to as Ito cells, fat-storing cell, star-like cells, or lipocytes, are highly specialized cells in the liver, responsible for the storage of most of the body’s retinoids (vitamin A or retinol and its metabolites). Together with Kupffer cells and liver sinusoidal endothelial cells (LSECs), HSCs are non-parenchymal liver cells and are located in the space of Disse, the perisinusoidal space between the parenchymal liver cells (hepatocytes) and a liver sinusoid [17]. Although total numbers of HSCs account for only 5–15% of the total liver cells [17,21,96], the vast majority of the hepatic retinoid stores are found in this cell population. Retinol is mainly stored as RE, the esterified form of retinol. These REs, predominantly retinyl palmitate in HSCs, are highly hydrophobic and are stored in the core of LDs. Vitamin A-storing stellate cells are mainly described for the liver, but morphologically similar cell types have been observed in a few other organs, e.g., pancreas and intestine. Yet, the involvement of these cells in pancreas and/or intestinal diseases are not as thoroughly studied as the HSCs. Amounts of REs found in LDs are reported to be 13–68% of the total neutral lipid content, depending on the dietary retinoid intake [97,98]. Furthermore, HSC LDs appear to be relatively large in size and small in number. Although diameters up to 8 μm are reported, most investigators report average LD diameters between 1.1 and 2.0 μm, depending on the animal, dietary status or imaging technique [99,100,101,102].

Upon various types of damage to the liver (e.g., caused by excessive hepatic lipid accumulation or viral insults), the quiescent and lipid-laden HSCs transdifferentiate into an activated, myofibroblast-like phenotype, devoid of the characteristic large and RE-positive lipid droplets. These activated cells produce significant amounts of extracellular matrix (ECM), a hallmark of fibrosis. Hence, the activation of HSCs is one of the major drivers of hepatic fibrosis. In line with the zonation in lipid accumulation observed in hepatocytes, activation of HSCs is heterogeneous [103] and primarily found in the periportal zone [104].

### 4.1. Lipid Droplet Dynamics in Activating HSCs

Although two activities that catalyze retinol esterification are reported, the predominant activity in quiescent HSCs is exhibited by the enzyme lecithin: retinol acyltransferase (LRAT). LRAT esterifies retinol utilizing the *sn*-1 fatty acid of PC. Mice lacking LRAT store hardly any REs in the liver [105,106,107]. By contrast, hepatic RE-levels are not affected in mice lacking Dgat1 (diacylglycerol O-acyltransferase 1), an enzyme that, besides its primary involvement in TAG-synthesis, also has the capacity to synthesize REs [108].

An early report showed that rats kept on a vitamin A-deficient diet for eight weeks did not have the large LDs that are so characteristic for HSCs [109]. Later, transmission electron microscopy revealed that also LRAT^-/-^ livers from mice lack these large LDs in HSCs [107], an observation that was confirmed by flow cytometry [110]. This lack of LDs is surprising as HSC lipid droplets do not solely contain REs, but also TAGs and CEs [101]. Indeed, studies of isolated LRAT^-/-^ HSCs under cell culture conditions show that these cells still have the intrinsic ability to synthesize small TAG/CE-containing LDs [111]. Why LRAT^-/-^ HSCs lack their large and RE-positive LDs in vivo remains to be elucidated.

A characteristic feature of HSCs is their ability to differentiate into a myofibroblastic cell type during liver injury/inflammation. During this process, RE-containing lipid droplets are degraded, while fibrillar collagen and growth factors are secreted. This “activation” process can be mimicked in vitro by culturing freshly isolated HSCs in plastic dishes containing medium with fetal bovine serum (FBS) [17]. This in vitro activation, most likely triggered by the combined stiffness of the dish [112] and pro-fibrotic components in the serum, results in the upregulation of typical activation markers such as α-smooth muscle actin (α-SMA) and collagen α1(I), accompanied by the loss of retinoids [17]. However, it should be noted that this activation procedure results in gene expression patterns that only partly matches those from activated HSCs in vivo [113] and obtained data should be interpreted with caution.

**Figure 3:**
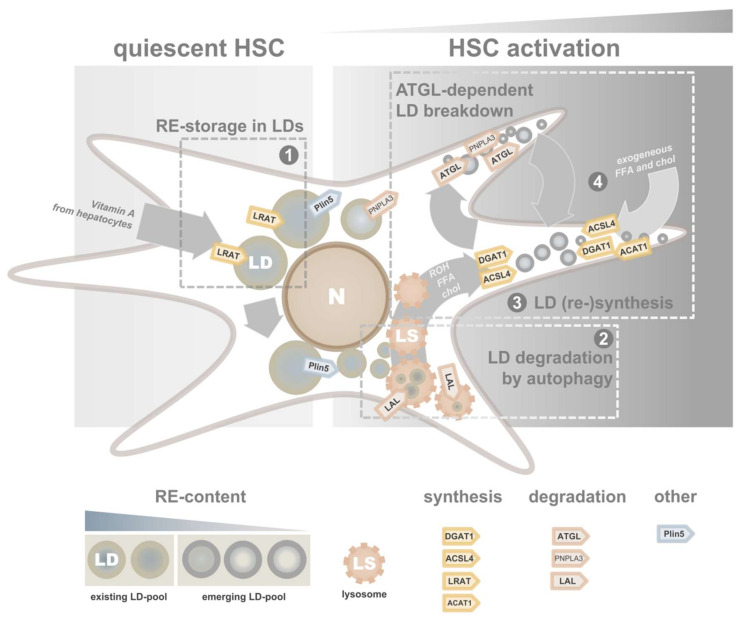
Lipid droplet dynamics in quiescent and activating hepatic stellate cells. HSCs have two distinct metabolic LD pools: “original” LDs, associated with quiescent HSCs (**left**) and “new” LDs that emerge during HSC activation (**right**). While “original” LDs (**1**) are predominantly degraded by lysosomes (**2**), “new” LDs are quickly recycled by repetitive synthesis by ACSL4 and DGAT1 (**3**) and degradation by neutral lipases (**4**)**.** ACSL4, long-chain fatty acid:CoA ligase 4; ATGL, adipose triglyceride lipase; chol, cholesterol; DGAT1, diacylglycerol acyltransferase 1; FFA, free fatty acid; HSC, hepatic stellate cell; LAL, lysosomal acidic lipase; LDs, lipid droplets; LRAT, lecithin retinol acyltransferase; LS, lysosome; N, nucleus; PLIN5, perilipin 5; RE, retinyl ester; ROH, retinol.

In HSCs isolated from rats, in vitro activation is accompanied by more than a 2-fold decrease in LD size 4 days [101], while numbers of LDs per cell dramatically increase. LD localization shifts from perinuclear to peripheral within a week (Figure 3). This change in morphology and intracellular localization is followed by a change in neutral lipid composition. Amounts of RE drops with more than 50% in 4 days, and after a week less than 20% of the original levels are still present. By contrast, the other neutral lipids found in LDs, CEs and TAGs show opposite dynamics. The increase in TAGs is driven by a pronounced increase in TAGs containing polyunsaturated fatty acids (PUFAs). Whether these described in vitro data reflect activating HSCs in vivo is unclear. Loss of REs was reported in fibrotic livers [102,114], but data about other neutral lipids in these cells are limited.

A number of morphological features of HSCs in fibrotic livers were studied by electron microscopy [115,116]. In both studies, liver biopsies of human patients with chronic hepatitis C were imaged. In healthy livers, large LDs were observed in HSCs. In livers with increasing fibrosis severity scores, numbers of LD-containing HSCs decreased [116]. Even in the most pronounced fibrotic stages, LDs did not disappear completely [115]. In addition, a comparison of HSCs isolated by two different FACS (Fluorescent Activated Cell Sorting) protocols underlined the relation between LD size and activation status: retinoid autofluorescence-sorted cells—associated with a quiescent state—display larger LDs than cells sorted by GFP under a collagen promotor—typically linked to a more activated state [100].

The counteracting dynamics of the different types of neutral lipids are intriguing: large vitamin A LDs appear to be replaced by smaller LDs containing polyunsaturated TAGs. Interestingly, early literature already proposed the existence of two types of LDs in HSCs [99,109,117]. Moreover, recent work from our group shows that HSCs cultured for a few hours have a non-uniform retinoid distribution over LDs. As revealed by retinoid autofluorescence, large LDs contain more vitamin A than smaller peripheral LDs [118]. These observations suggest the existence of distinct LD pools: an “original” LD pool and an emerging “new” LD pool [119]. We will further elaborate on their different metabolic characteristics in the next section.

### 4.2. Interfering by Targeting Specific LDs

The remarkable loss of lipid droplets in activating HSCs raises the question of how this loss is linked with activation. Do the cells need the content of the LDs for energy, membrane building blocks and/or signaling to facilitate activation, or are the big LDs a burden for the cell in a changing environment where its physiological role is different compared to its role in healthy livers?

#### 4.2.1. Targeting the “Original” LD Pool

Despite numerous reports that studied the relation between retinoids on the one hand and HSC activation and liver fibrosis on the other hand, the exact role of retinoids remains unclear. Whereas some early publications show that retinoids can suppress HSC activation and/or liver fibrosis [120,121,122,123,124,125], others have also reported the opposite [126,127]. These puzzling outcomes clearly indicate that the role of vitamin A and its numerous metabolites in liver injury is complex and that multiple factors are involved. Further, limitations of the culture and in vivo models, species differences, liver toxicity due to the administration of high concentrations of retinoids [128], and differences between endogenous and exogenous retinoids could affect outcomes. To overcome at least these last two considerations, a mouse model without endogenous stores of hepatic REs was studied [105,106,107]. Although the distribution and number of HSCs in these LRAT^-/-^ livers—almost devoid of hepatic REs—were not affected, HSCs lacked their characteristic large vitamin A-containing LDs. Subsequently, acute liver fibrosis was induced by bile duct ligation (BDL) or treatment with tetrachloromethane (CCl_4_). Remarkably, no signs of increased fibrosis were found in LRAT^-/-^ as compared to wild-type livers [107,110]. This finding argues that the presence of LDs in HSCs is not inversely correlated to fibrosis.

Another approach to interfere with the “original” LD pool to study its role in HSC activation is to block its degradation by inhibiting autophagy. Autophagy is a ubiquitous catabolic process that delivers molecules, cytoplasmic components and/or whole organelles to lysosomes for degradation [129]. A number of studies showed the involvement of autophagy in HSC LD degradation. First, in an immortalized mouse stellate cell line, JS-1, LDs increased after treatment with either autophagy inhibitor 3-methyladenine or lentiviral particles containing siAtg7, (impairing autophagy), as well as the expression of Plin2, an established LD marker [130]. Similar findings were described for primary murine and human HSCs [131]. Pharmaceutical inhibition of autophagy in these cells resulted in a significant dose-dependent increase in the number of large LDs as well as co-localization of BODIPY (lipid staining) and LC3B, a marker for autophagosomes [131]. Furthermore, inhibition of lysosomal acid lipase (LAL), a lipase activity in lysosomes and associated with RE degradation [132,133], was shown to attenuate LD degradation in HSCs, leading to accumulated vitamin A-positive structures in lysosomes [133].

Several groups report that blocking autophagy in HSCs affects activation and subsequent fibrogenesis. HSCs cultured in the presence of several autophagy inhibitors showed decreased expression of established activation markers [131]. Another group showed a decreased expression of α-SMA and collagen I in mouse HSC cell line JS-1 transduced with lentiviral particles containing siRNAs against Atg5 or Atg7 (impairing autophagy), indicating a diminished myofibroblastic HSC phenotype [130]. ATG-proteins are essential for autophagy, and knockdown results in suppression of the pathway. Inhibition of LAL results in decreased expression of the HSC-activation marker α-SMA [133]. The role of autophagy in HSCs was also confirmed in vivo. Making use of an HSC-specific Atg7-knockout (Atg7^F/F^-GFAP-cre), less severe signs of liver fibrosis in the knockout mice were observed after chemical induction of fibrosis [130]. Combined, these results indicate that interference of the “original” LD pool could be an effective target to suppress HSC activation (Figure 3). Obviously, it remains to be established whether the effect of autophagy on HSC activation is mediated by LD breakdown, or more specifically, the breakdown of LD components such as retinoids, CEs and/or TAGs into bioactive intermediates. Whether targeting liver autophagy could be used as a clinical approach remains to be elucidated. Despite the promising effects on HSC activation, inhibition of autophagy will also block LD degradation in hepatocytes and thus increase hepatic steatosis, as discussed earlier in this review.

#### 4.2.2. Targeting the “New” LD Pool

While the “original” LD pool predominantly consists of REs, the “new” pool is characterized by TAG species containing PUFAs. A key role in the synthesis of this pool is played by long-chain acyl-CoA synthetase 4 (ACSL4). This enzyme activates PUFAs to form fatty acid-CoA [134]. In contrast to other family members (ACSL1, ACSL3, ASCL5 and ACSL6), which have other specific fatty acid substrate preferences, both mRNA and protein levels of ACSL4 increased during HSC activation [135]. Immunofluorescence of HSCs revealed ring-like structures of ACSL4 surrounding lipid-dye LD540, indicating LD localization. Knockdown of ACSL by RNAi resulted in a decrease in PUFA-containing TAGs. Moreover, pharmacological inhibition of ACSL4 by the antidiabetic drug rosiglitazone shows a dose-dependent suppression of α-SMA expression in cultured HSCs [135].

Also, DGAT1 is involved in the formation of the “new” LD pool. This enzyme, together with its isoform DGAT2, is responsible for TAG formation by esterification of diacylglycerol with acyl-CoA. In contrast to DGAT2, knockdown of DGAT1 resulted in decreased synthesis of PUFA-containing TAGs in HSCs [136]. In addition, pharmacological inhibition of DGAT1 by T863 reduces the expression of activation marker α-SMA in rat HSCs. In murine HSCs, however, this was not the case [136], emphasizing species to species differences. The central role of DGAT1 over DGAT2 in HSCs is interesting, as pharmaceutical inhibition of the latter was shown to be effective in preventing lipid accumulation in hepatocytes (discussed earlier). Indeed, this treatment did not result in activation of HSCs [59]. It is unlikely that the ability of DGAT1 to synthesize REs plays a role during HSC activation as RE levels rapidly decline during this process and this activity is minimal in comparison to the RE-synthesis capacity of LRAT [111]. Hence, the dependence on incorporation of exogenous fatty acids during HSC activation (mediated by DGAT1) may provide an interesting therapeutic window to specifically target HSCs.

**Figure 4:**
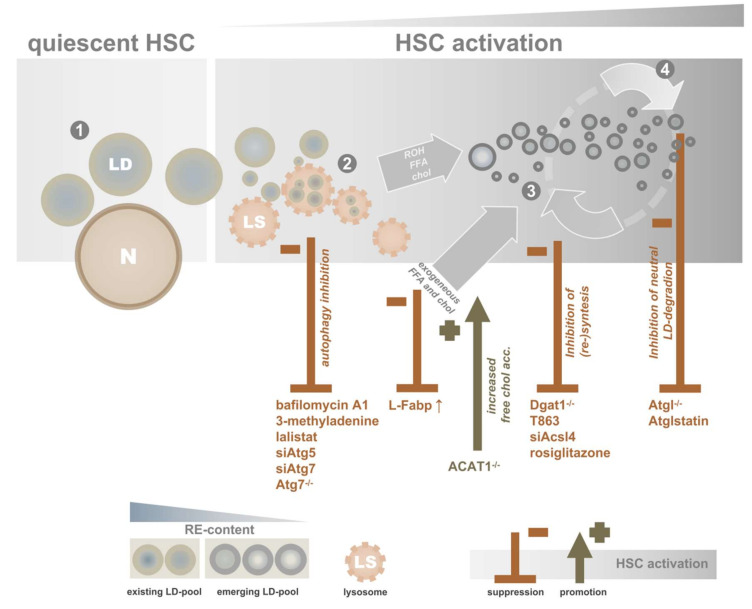
Lipid droplet homeostasis and HSC activation. Summary of targets and techniques that promote or suppress HSC activation as discussed in the main text. Numbers (**1–4**) refer to the stages described in Figure 3. si*X*, siRNA treated; *X*^-/-^, genetic knockout; *X* ↑, overexpression; ACAT1, acetyl-CoA acetyltransferase 1; chol, cholesterol; DGAT1, diacylglycerol acyltransferase 1; FAPB, FA-binding protein; FFA, free fatty acid; HSC, hepatic stellate cell; LDs, lipid droplets; LS, lysosome; N, nucleus; ROH, retinol.

In contrast to the “original” LD pool, the “new” LD pool appears to be less sensitive to lysosomal degradation. This pool is predominantly degraded by ATGL (also known as PNPLA2), which expression in HSCs was reported by several groups [136,137,138,139]. The inhibition of ATGL shows contradictory results. Although suppression of α-SMA expression was found in rat HSCs in the presence of ATGL-inhibitor Atglistatin [136,140], this effect was not observed in HSCs isolated from mice [136,138] (Figure 4). Whether this discrepancy can be explained by species differences or study design needs further investigation.

ATGL-dependent lipolysis is a highly regulated process, and several factors have been shown to affect lipase activity of ATGL. For instance, LD-protein Plin5 was shown to be involved in reduced lipolysis by biochemical ATGL-inhibition [141,142]. Interestingly, Plin5 is expressed in quiescent mouse HSCs, and its expression is decreasing during activation. Moreover, the exogenous expression of Plin5 suppresses activation and increased amounts of neutral lipids [143]. These observations fit in a model where the “original” LD pool is ATGL protected by Plin5, while the “new” pool, Plin5 depleted, might be more accessible for ATGL and its co-activators.

In addition to Plin5, fat-specific protein 27 (FSP27) was also shown to inhibit lipolysis by ATGL, both on the transcriptional level [144] and by biochemical interaction [145]. Strikingly, a study on FSP27 in HSCs showed very similar characteristics as compared to Plin5. First, initial expression in quiescent HSCs decreased during activation, and second, exogenous expression of FSP27 by lentiviral transductions decreased established activation markers in in vitro activated HSCs [146]. Taken together, these observations for both Plin5 and FSP27 in HSCs indirectly support the role of ATGL in HSC activation (Figure 3).

Several other activities, with PNPLA3 as the most prominent one, have been linked to neutral lipid degradation in HSCs. We refer the readers to Haemmerle and Lass [147] for more information about this topic.

#### 4.2.3. Targeting Cholesterol(esters)

Liver fibrosis is reported to be more severe in patients suffering hypercholesterolemia [148,149], a condition characterized by elevated levels of cholesterol in the blood. Moreover, free cholesterol (FC) and CEs were shown to accumulate in activated HSCs [101,148,149]. It was reported that FC accumulation in HSCs impairs lysosomal degradation of toll-like receptor 4 (TLR-4), thereby initiating a vicious cycle of increased sensitivity to TGFβ-induced activation and subsequent accumulation of cholesterol and TLR-4 protein [148,149]. In addition, it was suggested that conversion from FC to CE, stored in LDs, might be a target to break this loop; in HSCs lacking the predominantly expressed isoform of acetyl-coA cholesterol acyltransferase, ACAT1, FC levels were further elevated, resulting in increased expression of activation markers [150] (Figure 4).

Combined, the discussed data about LD dynamics in HSCs clearly show that the simple paradigm of “good” high-fat HSCs and fibrogenic low-fat HSCs is not the complete picture. To better acknowledge this subtlety in the screening of drugs targeting NALFD, systems containing both cell types are of interest.

## 5. Advanced In Vitro Models for Studying Liver Disease

Freshly isolated hepatocytes rapidly lose their biotransformation capacity, e.g., P450 CYP activity decreases to 30% within 24 h after isolation. For this reason, the establishment of a long-term culture of hepatocyte-(like) cells is instrumental in the study of cause–effect relations between hepatic lipid overload and lipotoxicity. Even more so, many more cell types other than hepatocytes (e.g., HSCs, Kupffer cells) play important roles NALFD [151]. As a result, designing in vitro models that include these cell types is essential to better understand the complex molecular details of the disease and to screen the efficacy of potential drugs. Only these type of models will allow to study the interactions between different cells, their feedback loops and substrate routing. Furthermore, when these model systems contain cells that are directly derived from the patient in question, screening for personalized medicine will come within reach.

Of course there is no better model to predict the response to particular drug treatments than testing on the person itself, but not desirable for many practical and ethical reasons. Cell lines (often tumor-derived) are very reductionistic model systems and have limited individual predictive value. Rodent models, often highly inbred with little genetic variation, are widely used for drug screening, but species-to-species differences hamper translation to the highly (genetically) variable human clinical practice. Recent developments in liver organoid technology combine the best of both worlds [152]. A practical definition describes an organoid as “an in vitro 3D-multicellular cluster derived from stem/progenitor cells, capable of self-renewal and self-organization, that recapitulates the function of the tissue from which it was derived” [153,154]. Organoids can be produced from individuals, and—especially in combination with other cell types—mimic real life to a certain functional degree. Mouse liver organoids were first described in 2013 by the *Clevers* group [155]. In the following years, liver organoids from humans, dogs, and rats have been described [156,157,158]. For cats, we reported a protocol to differentiate feline liver ductular stem cell-derived organoids into a hepatocyte-like phenotype with hepatocyte features such as albumin expression and CYP activity [159]. These organoids accumulate lipids when they are cultured in medium supplemented with exogenous fatty acids. Interestingly, an inverse correlation was observed between hepatocyte neutral lipid levels and cell survival [159]. This simplified model system allowed us to screen for various compounds which reduce neutral lipids in hepatocytes [160]. A limitation for the progression from a simplified NAFLD model (lipid-laden hepatocytes) to a simplified NASH model is the lack of profibrogenic stellate and inflammatory cells. To this end, a combination of hepatocyte-like cells (HepaRG) and stellate cells was introduced by the *Van Grunsven* group [161]. More recently, the same group generated HSCs from human iPS cells [162]. A co-culture of organoids with several patient-specific cell types was recently published by the *Takebe* group from Cincinnati, Ohio [163]. They reported an in vitro organoid model composed of hepatocyte-like, stellate-like, and Kupffer-like cells derived from iPS cells from healthy individuals and patients with steatohepatitis. These multicellular organoids acquired a steatohepatitis phenotype after fatty acid supplementation. The stiffness of these organoids increased, which is indicative of a fibrotic insult. Moreover, FGF19, the intestinal derived FXR-agonist, reverted some of the FFA-induced effects. This model represents the most advanced in vitro model for human liver fibrosis currently available and offers a promising opportunity to screen for antifibrotic drugs for personalized medicine. Several pharmaceutic targets have been suggested. For lipid-associated liver diseases, promising results were achieved after stimulation with the FXR-agonist obeticholic acid. The treatment improved lipid profiles of serum LDLs and other biochemical parameters such as γ-glutamyltransferase, alanine aminotransferase, FGF19 levels, and weight loss—all associated with beneficial effects for NASH patients [164,165,166,167,168]. Pharmaceutical targeting of the nuclear receptors Peroxisome Proliferator-Activated Receptor-α and -δ (PPAR-α and -δ), both playing central roles in the regulation of lipid metabolism, is currently under various stages of development [169,170,171]. For instance, the PPAR-α and -δ agonist GFT505/Elanfibror was shown to improve serum lipid profiles of patients [169,170,171].

## 6. Conclusions

“Love your liver, lower your lipids” could be a suitable slogan for a liver-disease-prevention campaign. However, just like most catchphrases, this simple one liner does not do justice to the immense complexity of lipid diversity and dynamics. A better understanding and appreciation of the diverse role of lipid metabolism in the many liver cell types will be key to prevent, halt, or cure liver fibrosis in the future. Lifestyle interventions such as lowering the caloric uptake and improving exercise are useful strategies to prevent or limit the risk for NAFLD [172]. However, pharmacological strategies to target NAFLD represent an attractive alternative in view of the increasingly large number of patients.
